# A Review of Novel Cancer Therapeutics and Current Research Trends

**DOI:** 10.1155/tswj/5056618

**Published:** 2025-11-12

**Authors:** Abimbola Mary Oluwajembola, Suleiman Zakari, Wisdom D. Cleanclay, Timothy Ayeni, Adewale Adebosoye, Olayinka S. Okoh, Joshua Folamade, Inalegwu Bawa, Olubanke Olujoke Ogunlana

**Affiliations:** ^1^Department of Biochemistry, College of Science and Technology, Covenant University, Ota, Ogun State, Nigeria; ^2^Cancer Genomics Laboratory, Covenant Applied Informatics and Communication Africa Centre of Excellence (CApIC-ACE), Ota, Ogun State, Nigeria; ^3^Department of Biochemistry, College of Medicine, Federal University of Health Sciences Otukpo, Otukpo, Benue State, Nigeria; ^4^Department of Biomedical Sciences, University of North Dakota, Grand Forks, North Dakota, USA; ^5^Department of Chemical Sciences, Faculty of Natural, Health and Applied Sciences, Anchor University, Ayobo, Lagos State, Nigeria

**Keywords:** cancer biology, cancer therapeutics, CAR-T, emerging therapies, gene therapy, PROTAC

## Abstract

The unchecked growth and spread of aberrant cells describe a widely diverse collection of disorders that collectively constitute cancer. Conventional therapies for cancer, including radiation therapy, chemotherapy, and surgery, have increased the chances of survival significantly in most patients. These traditional methods usually result in low tumor or tumor cell specificity, significant systemic toxicity, and the development of drug resistance. This review summarizes updates in cancer therapy, some of which include cutting-edge therapies represented by CAR-T therapy, targeted therapies, gene therapy, arginine-depriving therapy, mitochondria-targeted therapies, neutrophil-targeted therapies, and the latest PROTAC technology for proteolysis-targeting chimera. It has emphasized mechanisms underlying these new therapeutic strategies and their translational potential for treating human cancers. We further discuss, for each approach, the challenges, limitations, side effects, and delivery systems. The review proceeds with a dynamic change in the landscape of cancer research in biology, where machine learning and artificial intelligence are increasingly important to improve our understanding of the mechanisms of cancer and treatment responses. We also describe the potential of stem cell therapy, metabolomics, and novel drug delivery systems toward better patient outcomes. The paper pulls together some of the current research findings and results of clinical trials in new therapeutic developments and emerging areas of research that hold out exciting promises for the future progress of cancer treatment.

## 1. Introduction

One of the major causes of death globally is cancer, which is defined as a disorder in which aberrant cells proliferate out of control and infiltrate and destroy surrounding tissues [1]. Given that more than 18 million new cases are diagnosed annually worldwide, the burden of this disease process on humanity remains enormous [2]. Still, it knows no boundaries as it cuts across all demographics. Cancer is a heterogeneous disease consisting of so many subtypes that usually behave differently biologically and respond to treatment. The therapeutic strategies used today are surgical, radiation, and traditional chemotherapy, which have evolved over the past years for impressive improvements in survival rates from many malignancies. These, on many occasions, are associated with limitations like tumor or tumor cell nonspecificity, systemic toxicity, and development of resistance. Therefore, this involves the need for innovative treatment modalities to help provide better, targeted, and personalized care.

Cancer therapy has reached a momentous dawn where numerous therapies are in research and development. Emerging therapies for cancer refer to new and innovative approaches or treatments under relatively early stages of development and implementation [3]. Thus, the main goal of these therapeutic modalities is to improve existing treatments by increasing the specificity of action on cancer cells or by using the body's immune system against the disease [4]. Although immunotherapy, chemotherapy, and targeted therapy are the frequently used drug treatments for cancer, they have several adverse effects due to their limited specificity [5, 6]. Therefore, upcoming therapies are efforts to improve the efficiency of current treatments by directing them to cancer cells specifically and utilizing the immune system to fight the disease [5]. Such novel treatments are compulsory since they do not interfere with the normal working of healthy cells.

The emerging current therapies promise new effective ways to treat this complex disease. They can enhance patient outcomes and provide hope in people with few other treatment options, but they are not logical for patients who require organ, bone marrow, or stem cell transplants [7, 8]. Some of the new therapies are considered proteolysis-targeting chimeras (PROTACs), oncolytic viruses (OVs), nanoparticles, radiomics, ferroptosis-based therapy, chemodynamic therapy, ablation therapy, natural antioxidants, and sonodynamic therapy. Each possesses unique mechanisms for fighting cancer [9–13]. These new therapies, including CAR-T cells, targeted therapies, and gene therapy, are new options for standard treatment, exploiting specific molecular targets or enhancing the immune system's reaction to cancerous cells. For example, CAR-T cell therapy has been immensely successful in hematological malignancies, notably ALL or aggressive lymphoma, by engineering T cells to recognize and kill cancer cells expressing the target antigen specifically. Indeed, the mechanisms behind the targeted therapies all consider the tumor's unusual genetic and molecular characteristics, as in cases of breast cancer (HER2 positive) and melanoma with BRAF mutations, therefore permitting personalized modes of treatment with reduced damage to normal tissues. These new therapies make it critical to move beyond conventional therapy to address the changing landscape of cancer treatment. Riding the waves of progress in immunotherapy, precision medicine, and novel drug delivery systems allows us to enhance treatment efficacy, better patient outcomes, and reduce the toxicity of conventional therapies.

Given the evolving nature of cancer and the limitations of existing therapies, there is an urgent need for innovative treatment strategies. This mini-review attempts to present the current updates concisely in the evolution of emerging cancer therapeutics, such as targeted medicines, gene therapy, and immunotherapy, which have the potential to soon completely change how this illness is treated.

## 2. Recent Advances in Cancer Therapeutics

Recent progress in cancer therapeutics has given way to many newer strategies to enhance patient outcomes.

### 2.1. Gene Therapy

One of the quickly expanding cancer treatment options is gene therapy, which involves using various methods to change the genetic information inside the malignant cell. In cancer research, gene therapy is an exciting technique that uses genetic material manipulation to treat or prevent cancer [14–16]. It seeks to strengthen the body's defenses against cancer cells or repair or replace faulty genes that cause cancer cells to grow. Using both viral and nonviral vectors to introduce therapeutic genes into cancer cells is one method of gene therapy. The therapeutic gene will be delivered into cancer cells using the vector's specific targeting mechanism. Gene therapy works by specifically targeting tumor cells, protecting normal cells from cancer therapy side effects and boosting the immune system's capacity to identify and combat malignant cells [14]. One of the main strategies under this is Cas9/CRISPR Technology. This gene-editing tool makes it possible to change target genes in the genome with a great level of precision. Such changes can be done to rid the body of cancerous cells by inducing targeted destruction and inserting therapeutic genes.

**Figure 1:**
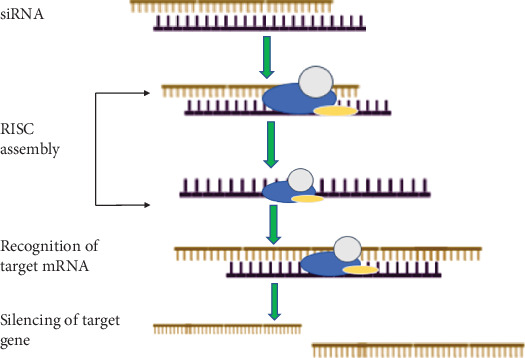
Mechanism of action of Small interfering RNAs (siRNAs) as RNA interference (RNAi) treatments. The general schematic shows the mechanism of siRNAs gene silencing. Double-stranded siRNA is incorporated by the RNA-induced silencing complex (RISC), which then discards one strand and uses the guide strand to direct RISC to complementary target mRNA. This causes mRNA to break and degrade, effectively silencing gene expression.

Research is being done on it to treat different kinds of cancer. Targeting cancer with gene therapy encompasses various strategies, such as (a) expressing a gene that causes apoptosis or makes the tumor more vulnerable to radiation or standard treatment; (b) introducing a wild-type tumor suppressor gene to make up for its dysregulation or disappearance; (c) employing an antisense (RNA/DNA) strategy to prevent an oncogene from being expressed; and (d) boosting tumor immunogenicity to encourage the recruitment of immune cells. The use of gene therapy to target the apoptotic machinery is justified by the fact that anomalies in the expression of pro- and antiapoptotic genes can result in apoptosis resistance in cancer cells, which is caused by faulty or absent apoptotic signaling. The introduction of genes encoding an inducer, mediator, or executioner of apoptosis is a widely used tactic in cancer gene therapy. According to recent research, RNA-based therapies have been identified as an emerging subclass of gene therapies that target cancer cells by altering their gene expression. These therapies include RNA interference (RNAi), antisense oligonucleotides, and mRNA-based vaccines, among others. Prostate and pancreatic cancers are among the cancers for which RNA-based treatments have demonstrated potential. Recently, RNAi, a method of gene silencing, has attracted more interest [17]. This biological process stops the expression of specific genes in disorders like cancer. It can be applied to improve the accuracy and efficiency of genetic therapy treatments. Small interfering RNAs (siRNAs) and microRNAs (miRNAs) are utilized in RNAi treatments to accelerate their activity while noncoding RNAs control cellular activities rather than translating into proteins. To stop the expression of certain genes, siRNA is loaded onto the RNA-induced silencing complex (RISC), which is made up of complementary messenger RNA nucleotide sequences, following transcription (Figure 1).

Higher silencing efficacy and stability are advantages of RNAi over conventional gene therapy. As a result, research on tumor gene therapy frequently uses it. For women, breast cancer is the most common type of cancer [18]. The development of distinct subtypes of breast cancer, the course of individual cases, and the reaction to therapy are all impacted by different gene expression profiles and genetic alterations. In this respect, a promising novel treatment option for breast cancer is gene therapy [19]. An octreotide (OCT)-targeted Lcn2 siRNA was loaded inside a PEGylated liposomal system in a study by Gote and his colleague for targeting metastatic breast cancer (MBC) cell lines MCF-7 and MDA-MB-231. The 152 nm sized, 0.13 PDI, 4.10 mV zeta potential liposomes had attained 69.5% entrapment and 7.8% loading efficiency [20]. OCT-Lcn2-Lipo increased MBC cell uptake, reduced Lcn2 mRNA to 55%–60%, and inhibited VEGF-A and endothelial cell migration, indicating potential antiangiogenic activity in MBC. Also, epigenetic treatment, an emerging therapeutic strategy that focuses on gene expression regulation without altering the DNA sequence, is sometimes combined with gene therapy for disease treatment. Inhibitors of DNA methyltransferase, BET inhibitors, and histone deacetylase are a few of these epigenetic treatments. Epigenetic medicines have demonstrated potential in the treatment of leukemia and lymphoma, among other cancers. Gene therapy in combination with epigenetic treatment allows for the repair of faulty genes and gene expression regulation. Target cells can be genetically modified by receiving genes, gene fragments such as siRNAs and miRNAs [14]. Although research conducted in clinical trials has demonstrated that this technique remains less destructive than conventional medicines [21], there are still obstacles to overcome; this includes the transport of oligonucleotide drugs to bodily regions that are inaccessible and the possibility of harmful side effects.

### 2.2. Immunotherapy

Immunotherapy allows the immune system to understand and kill the cancer cells. Prominent developments in this regard are as follows.

#### 2.2.1. Checkpoint Inhibitors

These checkpoint agents, which include inhibitors such as nivolumab and pembrolizumab—Opdivo and Keytruda—are directed against the immunologic checkpoints CTLA-4 and PD-1 that cancers use to evade immune detection. A way to block these checkpoints can enhance a T-cell's potential to fight cancer cells. These checkpoints are natural regulatory mechanisms that stop the immune system from targeting healthy cells. By inhibiting these checkpoints, the drugs enhance the ability of T lymphocytes to identify and combat cancer cells. Checkpoint inhibitors are effective against various malignancies, such as bladder, lung, and melanoma. Programmed death-ligand 1 (PD-L1) inhibitors, such as atezolizumab (Tecentriq and Tecentriq Hybreza), avelumab (Bavencio), and durvalumab (Imfinzi), target the PD-L1 protein on cancer cells to disrupt immune suppression. Ipilimumab (Yervoy) and tremelimumab (Imjudo), which are CTLA-4 inhibitors, obstruct early T-cell inhibition, thereby promoting immune activation. These agents have transformed the treatment paradigm by inducing sustained responses in patients with advanced or refractory disease [22]. In a recent study, HSC was used with PD-1 antibodies-decorated platelets to treat recurrent leukemia in mice [23]. Due to the tumor microenvironment, the HSCs moved to the bone marrow and produced PD-1 antibodies, which increased activated T-cell responses and raised survival rates.

#### 2.2.2. Chimeric Antigen Receptor (CAR) T-Cell therapy

**Figure 2:**
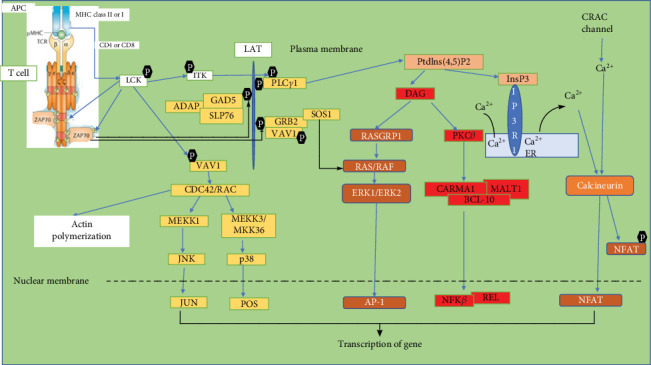
The CAR T cell signaling pathway. The pathway involves the following steps: (1) Recognition of target antigen: A specific antigen on the surface of a target cell is recognized and bound by the chimeric antigen receptor (CAR) on the surface of CAR T cells. (2) Activation of CAR: The binding of the CAR to the target antigen activates the CAR, leading to the phosphorylation of intracellular domains and recruitment of signaling molecules. (3) Signal transduction: The activated CAR triggers downstream signaling cascades, resulting in the activation of various kinases and transcription factors. (4) Cytokine release: The activated CAR T cell releases cytokines, like interferon-gamma and interleukin-2, which help to recruit and activate other immune cells. (5) Target cell killing: The activated CAR T cell releases cytotoxic molecules, such as perforin and granzyme, that trigger programmed cell death in the target cell.

CAR T-cell therapy, or treatment with CAR T-cells, involves using genetically modified T-cells to specifically target and eliminate cancer cells [24]. This kind of immunotherapy has shown potential when treating certain nonsolid malignancies, such as acute lymphoblastic leukemia (ALL) and non-Hodgkin lymphoma [25]. To generate the CAR T-cells, the T-cells are first taken out from the blood of the patient, after which they are genetically engineered to express the CARs before being introduced again by infusion into the patient's bloodstream to locate and eliminate cancerous cells [26, 27]. CAR-T cells are programmed to recognize and adhere to specific antigens on the surface of cancer cells, causing the T-cells to activate and destroy the cancer cells. Successes have been recorded in the use of CAR T-cells in clinical trials against different forms of cancer [26]. For example, in clinical trials involving persons with B-cell ALL who have relapsed or are not responding, treatment with CAR-T cells targeting the CD19 antigen resulted in remission rates of up to 94% [28]. Clinical trials using CAR-T cell treatment for multiple myeloma and non-Hodgkin lymphoma have demonstrated similar success [29, 30]. Examples of approved CAR-T therapies include tisagenlecleucel, also known as tisa-cel (Kymriah), approved for both pediatric and young adult B-cell ALL and adult diffuse large B-cell lymphoma and follicular lymphoma; axicabtagene ciloleucel, also known as axi-cel (Yescarta), which was approved for treating large B-cell lymphoma and follicular lymphoma; brexucabtagene autoleucel, also known as brexu-cel (Tecartus), approved for the treatment of adult B-cell ALL and mantle cell lymphoma. Lisocabtagene maraleucel, also known as liso-cel (Breyanzi), approved for follicular lymphoma, large B-cell lymphoma, mantle cell lymphoma, and chronic lymphocytic leukemia, while idecabtagene vicleucel, also known as ide-cel (Abecma), and ciltacabtagene autoleucel, also known as cilta-cel (Carvykti), had been approved for multiple myeloma in relapsed patients. Obecabtagene autoleucel, also known as obe-cel, was approved for B-cell ALL in adults [31, 32]. Remarkable success has been witnessed with the use of these CAR-T cell therapies in clinical trials, with many patients achieving complete remission [28–30]. This therapy represents potential progress in cancer therapy, and the development of new and more effective cancer treatments may be the outcome of progressive research in this field [24, 25]. As depicted in Figure 2, CAR-T cells are designed to express CARs that are made up of a domain that binds antigen, normally derived from a monoclonal antibody, linked to a signaling domain derived from the TCR complex. Upon binding to their target antigen, CAR-T cells activate the TCR signaling pathway, leading to T cell activation and proliferation, synthesis of cytokines, and ultimately, cancer cell killing. Another important pathway in CAR-T cell therapy is the cytokine signaling pathway. Upon activation by their target antigen, cytokines like interleukin-2 (IL-2) and interferon-gamma (IFN-*γ*) are secreted by CAR-T cells [33]. Cytokines are small proteins with significant roles in regulating the growth and activity of other immune system and blood cells. Interleukins and interferons help to boost anticancer activity by transmitting signals that activate immune cells. Chemokines, a certain category of cytokines, promote inflammation or immune cell recruitment, thereby enhancing immune tumor suppression.

Despite its promise, CAR-T therapy is highly expensive and comes with serious adverse effects such as cytokine release syndrome (CRS) and neurotoxicity (ICANS), requiring strict monitoring and control. Long-term consequences such as cytopenias and infection also contribute to the adverse effect of this therapy. Additionally, the very high price, sometimes more than $400,000 for each course of treatment, significantly restricts access to the majority of patients that could benefit from this therapy [34, 35].

## 3. Targeted Therapies

Targeted therapies are a class of drugs designed to specifically address molecular or genetic aberrations present in cancerous cells. These therapies, often directed by biomarkers, include small molecule inhibitors and immunotherapies such as antibody–drug conjugates (ADCs) and monoclonal antibodies [36, 37]. Targeted therapies have demonstrated effectiveness in the treatment of different cancers, such as breast, melanoma, and lung cancer, with biomarkers playing a key factor in treatment decisions. One example of a monoclonal antibody therapy is trastuzumab, which targets the HER2 protein overexpressed in breast cancer cells. In order to destroy cancer cells, trastuzumab binds to the extracellular domain of HER2, blocking downstream signaling pathways and triggering antibody-dependent cellular cytotoxicity (ADCC). Another example is cetuximab, which targets the epidermal growth factor receptor (EGFR) overexpressed in colorectal cancer cells. Cetuximab binds to the EGFR extracellular domain, blocking downstream signaling cascades and inducing ADCC. Another kind of targeted treatment is called a small molecule inhibitor, which functions by blocking particular enzymes or signaling pathways that are crucial for the development and survival of cancer cells. One well-known medication that targets the BCR-ABL fusion protein in the management of chronic myeloid leukemia (CML) cells is imatinib. By attaching to the BCR-ABL protein's ATP-binding site, imatinib inhibits downstream signaling cascades and causes cancer cells to undergo apoptosis. ADCs such as brentuximab vedotin (Adcetris) target the CD30 antigen on lymphocytes and are attached to the chemotherapy drug MMAE, and ado-trastuzumab emtansine (Kadcyla, TDM-1) targets the HER2 protein and is attached to the chemotherapy drug DM1. This therapy combines antibodies with chemotherapeutic agents, bringing cytotoxic drugs directly to cancer cells, thereby enhancing effectiveness while reducing systemic toxicity [38–40]. Immunotherapy is a new form of targeted therapy that fights cancer by stimulating the body's immune system. It utilizes the immune system of the body to identify and fight against cancer cells. It includes therapies such as adoptive cell transfer, cancer vaccines, and checkpoint inhibitors. Checkpoint inhibitors, namely, pembrolizumab and nivolumab, have proven to be effective in the treatment of many cancer types. Numerous malignancies, such as melanoma, bladder, and lung cancer, have been successfully treated using immunotherapy. Targeted therapy for cancer essentially targets specific substances and biological mechanisms that are involved in the development and spread of cancer cells. Overall, targeted therapy has revolutionized cancer treatment by making a more personalized and effective approach to the treatment of cancer available. Targeted therapy is anticipated to become more important in the treatment of cancer as new molecular targets are identified and innovative treatments are created.

### 3.1. Arginine-Depriving Enzyme Therapies

**Figure 3:**
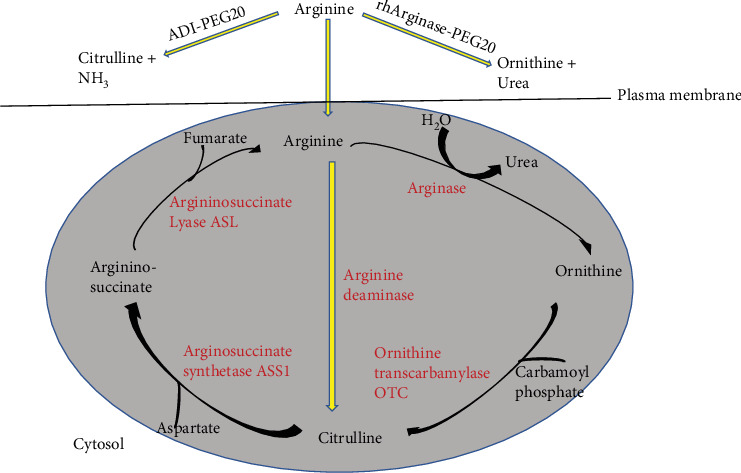
Arginine deprivation therapy [45]. The amino acid arginine is essential and required for cell growth and protein synthesis; therefore, arginine-depriving enzymes like arginase and arginine deiminase can selectively target tumor cells that have a high demand for arginine.

A form of cancer treatment called arginine-depriving enzyme therapy takes advantage of the tumor's arginine-dependent growth and survival [41–44]. The amino acid arginine is required for both the growth of cancer cells and the creation of proteins. In comparison to normal cells, cancer cells require more arginine and are unable to produce it on their own. Therefore, they depend on the uptake of arginine from the surrounding microenvironment. A novel approach to treating malignancies lacking argininosuccinate synthetase 1 (ASS1) expression is arginine deprivation treatment (Figure 3). For citrulline to be converted to arginine, the enzyme ASS is essential [46]. One innovative therapeutic approach for the treatment of cancer is arginine deprivation. It causes caspase-dependent apoptosis and autophagy in susceptible tumor cells, particularly in those that lack arginine succinyl synthetase [47]. ASS1-negative cancers are thought to respond better to arginine deprivation therapy than tumors with low ASS1 expression [48]. Deprivation of arginine can change signaling pathways. For example, it affects the activation of mTOR and p70S6K, which in turn causes the PI3K/Akt pathway to become inactive [48]. Arginine deprivation has garnered substantial support as a novel and potentially safe antimetabolite treatment used to address a variety of challenging cancers characterized by a critical arginine deficiency [49]. It is effective against different types of tumors, including melanoma, acute myeloid leukemia (AML), and prostate cancer [49].

The therapy also involves the use of enzymes, such as arginine deiminase (ADI) and pegylated ADI, to deplete the levels of arginine in the tumor microenvironment (Figure 3) [42]. By depriving cancer cells of arginine, the therapy induces cell death and inhibits tumor growth. Several studies have demonstrated the efficacy of arginine-depriving enzyme therapy in treating a range of cancer types, such as prostate, melanoma, hepatocellular carcinoma, and leukemia. Clinical trials are being conducted to assess the therapy's safety and effectiveness in treating various cancer types. The enzyme arginase decreases the amount of arginine available for the proliferation of cancer cells by converting it to ornithine and urea. Research has demonstrated that arginase prevents prostate cancer cells from growing in vivo and in vitro [50]. Another arginine-depriving enzyme that causes arginine to be converted to citrulline is ADI, which lowers the availability of arginine in cancer cells. It has been demonstrated that ADI prevents melanoma cells from growing and makes them more susceptible to chemotherapy [51]. Several clinical trials are being conducted to evaluate the safety and effectiveness of treatments that deplete arginine from cancer cells. A pegylated form of ADI known as ADI-PEG 20 has been assessed in clinical trials for effectiveness against various types of cancer, including hepatocellular carcinoma, melanoma, and mesothelioma, with promising results [49, 51]. In summary, treatment for cancer using arginine-depriving enzyme therapy is a promising strategy, especially for tumors that have a high arginine demand. Clinical trials are being conducted to assess the safety and effectiveness of these treatments, but more research is necessary to ascertain the most effective method to utilize them and which patients might benefit the most from them. Overall, the prognosis and survival of patients with metastatic prostate cancer and other cancer types can be improved by using arginine deprivation therapy, a novel and promising cancer treatment strategy.

### 3.2. Mitochondrion as an Emerging Therapeutic Target

**Figure 4:**
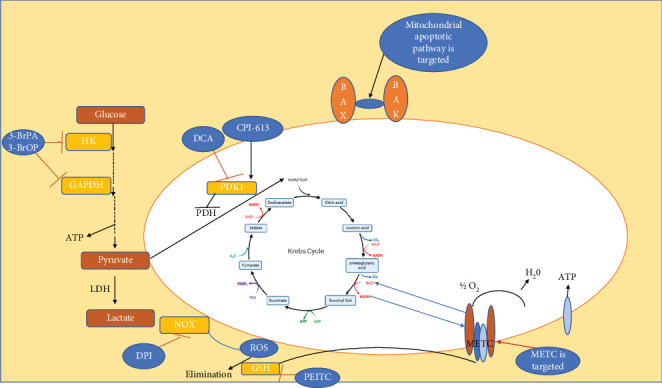
Mitochondria as special target. Treatment for cancer using mitochondria-targeted therapy is a promising strategy, as it targets a critical cellular organelle with multiple functions in cancer cell survival and proliferation [52, 58].

Treatment for cancer using mitochondria-targeted therapy is a promising approach since the role of mitochondria in the generation of cellular energy, reactive oxygen species (ROS) generation, and cell death through apoptosis is pivotal [52–55]. Mitochondria-targeted drugs have been developed to deliver drugs to mitochondria in a selective manner and, as a result, boost their effectiveness and reduce off-target effects [56]. One such example is the mitochondria-targeted antioxidant MitoQ, which has indicated promise in preclinical research as a potential treatment for cancer [56]. In addition to antioxidants, inhibitors of the electron transport chain (ETC) and mitochondrial fission/fusion proteins are further medicines that target the mitochondria. It has been demonstrated that ETC inhibitors, like metformin, prevent mitochondrial respiration and cause cancer cells to die [57]. Mitochondrial fission/fusion proteins, which are involved in regulating mitochondrial dynamics and function, have also become prospective targets of focus for cancer treatments (Figure 4). More research is necessary to completely comprehend the mechanisms of action and potential drawbacks of these medicines.

### 3.3. Neutrophils as Emerging Therapeutic Targets

Neutrophils may be a viable therapeutic target in the treatment of cancer since evidence exists that indicates that they are essential to the growth and metastasis of cancer [59, 60]. As a type of white blood cell, neutrophils are essential to the innate immune system and are vital in protecting the body against infections. However, in the context of cancer, neutrophils can promote tumor growth and progression by producing proinflammatory cytokines, angiogenic factors, and enzymes that degrade the extracellular matrix (ECM) [61]. Neutrophils can also support premetastatic niche formation, a process by which cancer cells prepare a distant site for colonization; neutrophils can create a favorable environment for tumor growth and angiogenesis in this niche, which allows cancer cells to establish new tumors at distant sites [62]. Several strategies are being explored to target neutrophils in cancer treatment [63]. One approach is to stop the chemokines such as CXCL1, CXCL2, and CXCL8 (IL-8) that draw neutrophils to the tumor site to prevent their recruitment and usual overexpression in the tumor microenvironment [64]. Another strategy is to target neutrophil-derived enzymes that promote cancer cell invasion and migration, such as matrix metalloproteinases (MMPs) [65]. Furthermore, recent research has indicated that immunotherapy, which includes inhibitors of immune checkpoint and CAR T cell treatment, can target neutrophils [66]. In preclinical models, CAR T-cells that target neutrophil surface markers produced promising results in suppressing tumor growth and prolonging survival [66]. It has also been demonstrated that immune checkpoint inhibitors, such as anti-PD-1 and anti-CTLA-4 antibodies, increase neutrophil-mediated anticancer activity [67]. Targeting neutrophils is an emerging strategy in cancer treatment. By inhibiting neutrophil recruitment and activation or enhancing their antitumor activity, it is possible to suppress tumor growth and prevent metastasis.

### 3.4. PROTAC Technology

An emerging field of cancer therapeutics known as PROTAC uses small molecules to target the degradation of particular proteins by the ubiquitin-proteasome system (UPS) [12, 68–70]. Two small molecules make up the PROTAC technology: one targets the desired protein, while the other recruits an E3 ubiquitin ligase to the protein complex, causing its ubiquitination and eventual destruction [71]. Comparing PROTAC technology to conventional small molecule inhibitors reveals several benefits. First off, because of their intracellular location or absence of small molecule binding sites, proteins that are typically thought to be “undruggable” with small molecules can be targeted by PROTACs. Secondly, PROTACs can induce a more complete degradation of the target protein compared to traditional inhibitors, which may only inhibit protein activity. Thirdly, PROTACs have been shown to have a prolonged effect on target protein degradation, even after the small molecule has been cleared from the system. A highly promising use of PROTAC technology is in the treatment of cancer [72]. In preclinical studies, PROTACs have been shown to effectively target several proteins that are implicated in cancer pathogenesis, including oncogenic transcription factors, signaling kinases, and protein–protein interaction domains. For instance, ARV-110, a PROTAC that targets the androgen receptor for degradation, is currently being investigated in clinical trials and has demonstrated encouraging outcomes in preclinical models of prostate cancer [73].

Additionally, PROTAC technology may be able to circumvent drug resistance mechanisms that are frequently seen in cancer treatment. For example, in HER2-positive breast cancer, resistance to traditional small molecule inhibitors can occur due to amplification of the HER2 gene, leading to an overexpression of the protein. However, a PROTAC that targets HER2 for degradation has been shown to overcome this resistance mechanism by inducing the degradation of HER2 even in cells with high levels of protein expression [74]. Because PROTAC targets proteins that are previously inaccessible, it has the potential to transform cancer treatment altogether, inducing complete degradation of target proteins and overcoming drug resistance mechanisms [75].

## 4. Promising Avenues for Cancer Research and Therapeutics

### 4.1. Nanomedicine

Nanomedicine is an emerging field that uses nanoparticles and other nanoscale materials to diagnose, treat, and prevent disease [76]. In cancer research, nanomedicine offers several potential applications, including targeted drug delivery, imaging, and sensing. It has the capacity to transform cancer research by improving the accuracy and effectiveness of cancer diagnosis, treatment, and monitoring [5, 77]. The capacity of nanomedicine to specifically target cancer cells while preserving healthy cells is one of its key benefits. This is achieved by engineering nanoparticles with specific surface properties that allow them to bind to cancer cells. Once the nanoparticles are bound, they can release drugs or other therapeutic agents directly into the cancer cells, minimizing side effects [78, 79].

Nanoparticles can also be used for cancer imaging, allowing for earlier and more accurate diagnosis. For example, magnetic nanoparticles can be used to create contrast in magnetic resonance imaging (MRI), enabling the detection of small tumours that may not be visible with other imaging techniques [80, 81]. In addition to drug delivery and imaging, nanomedicine can also be used for cancer sensing and monitoring. Nanoparticles can be engineered to detect specific biomarkers associated with cancer, such as proteins or genetic markers. By monitoring changes in these biomarkers over time, doctors may be able to detect cancer earlier and track the effectiveness of treatment [82, 83].

### 4.2. Extracellular Vesicles (EVs)

Cells release tiny, membrane-bound particles into the extracellular environment called EVs. These vesicles play a critical role in cell-to-cell communication, and their contents can offer crucial information about the health and activity of the cells that release them [84, 85]. EVs are able to transport a large range of biomolecules, including proteins, nucleic acids, and lipids, which can provide insight into the state of the cell that produced them.

In cancer research, EVs are emerging as a promising area of investigation with potential applications in diagnosis, prognosis, and therapy [86, 87]. EVs have been shown to carry specific biomarkers that are associated with disease progression and response to therapy [5, 88]. Therefore, by analyzing the contents of EVs, researchers may be able to develop more accurate diagnostic tests and prognostic indicators for cancer. EVs also have potential applications in cancer therapy. EVs can be engineered to deliver therapeutic agents, such as siRNAs or anticancer drugs, directly to cancer cells. Because EVs are derived from the patient's cells, they are less likely to be recognized as foreign by the immune system and can potentially reduce the risk of adverse effects associated with traditional drug delivery methods [89–91]. In addition to drug delivery, EVs also play an immunomodulatory role in cancer therapy. Recent studies have shown that EVs can stimulate the immune system and promote antitumor responses, potentially enhancing the effectiveness of cancer immunotherapy [91, 92].

### 4.3. Radiomics and Pathomics

Radiomics is the process of quantifying tumor properties at a high throughput level through the analysis of medical images. Pathomics, on the other hand, involves the generation and characterization of high-resolution tissue images [5]. Radiomics is a rapidly developing field in cancer research that uses advanced imaging techniques, such as computed tomography (CT), MRI, and positron emission tomography (PET), to extract and analyze quantitative features of tumors. These features can provide valuable information about the tumor's size, shape, texture, and heterogeneity, which can be used to predict the tumor's behavior and response to treatment [5]. Radiomics has been used to predict patient survival, differentiate between benign and malignant tumors, and determine which patients would likely gain from chemotherapy [93, 94]. Pathomics has shown promise in a range of cancer types, including breast cancer, lung cancer, and prostate cancer. For example, in breast cancer, pathomics has been used to identify new subtypes of the disease and to predict patient outcomes based on the genetic signature of the tumor [95].

### 4.4. OVs

A novel family of therapies for cancer known as OVs preferentially proliferate within and destroy cancer cells, while sparing normal cells. OVs are a potential cancer therapeutic strategy as they can also activate the immune system to combat cancer cells [10, 96]. Recent findings have shown the potential of OVs as a powerful tool in cancer management. One of the most promising OVs is the herpes simplex virus type 1 (HSV-1). HSV-1 has been modified to selectively replicate in and kill tumor cells while sparing normal cells. A recent study showed that an oncolytic HSV-1 virus, T-VEC, improved overall survival in patients with advanced melanoma [97]. Another study demonstrated that T-VEC combined with pembrolizumab, an immune checkpoint inhibitor, showed a higher response rate and longer progression-free survival in patients with advanced melanoma compared to pembrolizumab alone [98]. Other OVs that have demonstrated potential in both preclinical and clinical research include adenoviruses, reoviruses, measles viruses, and vaccinia viruses. For instance, a recent study showed that an oncolytic adenovirus, DNX-2401, improved survival in patients with recurrent glioblastoma [99]. Another study demonstrated that a combination of reovirus and chemotherapy improved survival in patients with metastatic pancreatic cancer [100]. OVs have also been shown to be effective in combination with other cancer treatments such as chemotherapy and radiation therapy. A recent study showed that combining an oncolytic vaccinia virus with radiation therapy enhanced survival in patients having advanced solid tumors [101]. Recent findings have demonstrated the potential of OVs as a powerful tool in cancer management. OVs have shown promise in preclinical and clinical studies and have shown effectiveness in combination with other cancer therapies.

## 5. Emerging Areas of Cancer Biology Research

Cancer biology research spans a broad spectrum with numerous emerging fields focused on comprehending how cancer develops and progresses. These new areas encompass artificial intelligence (AI) technologies for predicting cell and population growth, hybrid modelling approaches that blend data-driven and mechanistic methods, interpretable AI models for cancer initiation and progression, morphology-based molecular characterization and cell dynamics, the role of ECM proteins, matrix remodeling enzymes, and cytokines in guiding cell migration, the ECM's impact on stem cell maintenance, tumor initiation, progression, and therapy resistance, advances in cancer metabolism, including the functions of metabolic intermediates, the link between body balance and cancer cell biology, mechanisms connecting obesity to cancer, the adaptability of cancer cells, and the effects of diet and fasting on cancer initiation. These emerging areas of research are crucial in identifying novel targets and designing new strategies for the prevention and treatment of cancer. We will highlight some of the emerging areas of cancer biology research. Cancer biology research is a vast field with many emerging areas of study. Some of these areas include:

### 5.1. AI and Machine Learning (ML)

AI and ML are rapidly evolving fields that have shown significant potential in cancer diagnosis, prognosis, and treatment [8, 102–104]. These technologies can analyze vast datasets and identify patterns and trends that are imperceptible to the human eye. AI and ML can also be utilized to create personalized treatment plans for cancer patients tailored to their specific genomic profiles. Possessing the capacity to examine vast quantities of data and spot trends, AI and ML can assist in early detection, diagnosis, and personalized treatment plans for cancer patients. One example is the use of AI in medical imaging, such as mammography and MRI scans, to detect early signs of cancer that may not be detectable by the naked eye [103]. This can lead to earlier diagnosis and more effective treatment. A recent study showed the use of ML applications in cancer management and discussed the potential of ML to improve prostate cancer diagnosis and treatment [105]. Yoo and his team created SCORPIO, an immune checkpoint inhibitor cancer patient survival-predicting ML model, utilizing regular blood tests and clinical information and avoiding advanced genomic analysis. According to this study, SCORPIO outperformed tumor mutational burden, maintaining superior predictive performance for predicting immune checkpoint inhibitors' outcome, even after validation [106]. ML can be used to analyze medical images, such as MRI and histopathological images, to aid in the detection and diagnosis of prostate cancer. ML can also be used in surgical robotics to improve surgical precision and autonomy. The article provides examples of ML applications in each of these areas and discusses the potential of ML to improve prostate cancer diagnosis and treatment. In addition, ML can be used to analyze patient data, including genomic information, to create individualized treatment plans. This can lessen adverse effects and enhance therapy outcomes.

The potential use of AI and ML techniques in the clinical management of prostate cancer patients was analyzed recently [107]. The authors analyze published studies on AI and ML techniques related to prostate cancer, including digital pathology, genomics, and treatment prediction. AI is transforming the healthcare industry, particularly in the field of prostate cancer diagnosis. Digital pathology is being augmented by AI to assist researchers in analyzing bigger data sets, leading to faster and more precise diagnoses of prostate cancer lesions [107, 108]. Moreover, AI has shown remarkable correctness in detecting prostate cancer and in prognosis prediction when applied to diagnostic imaging. They also highlighted the potential of AI and ML techniques in improving the accuracy and efficiency of prostate cancer diagnosis and treatment prediction [107]. Furthermore, the use of AI in cancer research to integrate multi-omics data is fast-growing, advancing the field of modern biology. Complex correlations between genomic, transcriptomic, proteomic, and epigenomic profiles can be revealed by ML algorithms, leading to more precise tumor classification, prognosis prediction, and treatment selection. Understanding tumor heterogeneity at the systems biology level and discovering new biomarkers have been made possible by AI-supported multi-omics platforms [109–111]. Although AI and ML have proven to be highly promising in the field of oncology, there is a need for their validation and regulation among other limitations and challenges of their implementation in clinical practice.

#### 5.2. Stem Cell Therapy

Stem cell is an emerging area of cancer research that shows great promise in the treatment of various types of cancer. This therapy employs stem cells that can differentiate into different cell types to regenerate and repair damaged tissues in the body [112–114]. Different types of stem cells have been used in cancer treatment, depending on their unique abilities. The transplantation of human stem cells has proven to be a successful method for treating blood cancers such as leukemia, multiple myeloma, and lymphomas. They have varying abilities in terms of proliferation, migration, and differentiation, which are important factors in determining their use in cancer treatment. Embryonic stem cells (ESCs) can develop into different cells excluding those in the placenta, but their application in clinical trials is limited due to ethical concerns. Adult stem cells (ASCs), such as mesenchymal and hematopoietic stem cells and neural stem cells, are commonly used in cancer treatment because they can produce many specialized cell types found in tissues and organs. Cancer stem cells (CSCs), in contrast, are immature progenitors of tumor cells that are produced by epigenetic mutations in normal stem cells or precursor/progenitor cells [114]. CSCs were discovered in leukemia in 1994 and have been seen as a hopeful target for cancer treatment [115]. These cells are implicated in numerous aspects of tumor malignancies, including recurrence, metastasis, heterogeneity, and resistance to multiple drugs. The unresolved issues of high recurrence and mortality rates in cancer are closely related to the biological features of CSCs [114, 116, 117].

In cancer treatment, stem cell therapy is used to replace the bone marrow of patients undergoing chemotherapy or radiation therapy that is damaged or destroyed. This is because these treatments can damage healthy cells in the body, including the bone marrow, which produces blood cells. The use of engineered neural stem cells (NSCs) is becoming a hopeful approach to treating cancer [118]. By engineering NSCs with different therapeutic agents, preclinical studies have shown tumor reductions of 70%–90% [118]. The use of tumoricidal NSC therapy is creating new opportunities for cancer treatment. These cells can target tumors and deliver drugs to cancerous areas that are difficult to reach with traditional methods like surgery, chemotherapy, and radiotherapy. Stem cell therapy can also be used to deliver targeted therapies directly to cancer cells, which may diminish adverse effects and improve therapeutic efficacy. This is achieved by engineering stem cells to produce specific proteins that can target cancer cells and deliver therapeutic agents directly to them. While stem cell therapy is still in its early stages of development, it holds great promise for the future of cancer treatment. Ongoing experiments focus on how stem cells signal tumor growth and metastasis in specific situations, as well as identifying the best strategy for engineering stem cells. Due to the complex and immunosuppressive nature of solid tumor microenvironments, combining stem cell therapy with other treatments like ADCs, immune cytokines, and inhibitors of immune checkpoint may offer better results in fighting cancer and preventing recurrence. However, previous studies have shown that stem cells, especially mesenchymal stem cells (MSCs), may have protumoral effects in some situations, despite their potential for regenerative treatment and their capacity to settle in tumor sites. Through processes like immunosuppression, proangiogenic and growth factor release, tumor microenvironment alteration, and metastasis, MSCs have been demonstrated to accelerate the growth of tumors [119–121]. Therefore, investigations into long-term safety and effectiveness are still ongoing.

## 6. Challenges and Future Directions

Despite the developments in cancer therapeutics, several concerns still exist:

*Overcoming drug resistance*: There is a need to develop strategies that counteract resistance mechanisms to enhance the possibility of successful treatment. Understanding the specific biology underlying resistance will inform the design of more efficient therapies.

*Improved efficacy with reduced side effects*: Further research in the field of oncology is necessary to enhance the therapeutic index of treatments to be both effective and tolerable.

*Integrating new therapies into clinical practice*: Guidelines pertaining to integrating new therapies into routine clinical care will be of paramount importance in optimizing the management of patients for the best possible outcomes.

## 7. Conclusion

Emerging areas of cancer biology research are essential in identifying new targets and designing novel cancer prevention and treatment strategies. These research areas are rapidly evolving and hold great promise for improving cancer patients' outcomes. Spanning from immune checkpoint inhibitors to CAR-T cell therapy, and from monoclonal antibodies to OV therapy, these strategies manifest huge potential for improving patient survival in a wide range of cancer diseases. Further investigation in this area brings combination therapies and patient-specific designs, based on their respective immune profiles, closer to realization. While challenges persist, notably variability of patient responses and potential side effects, the continued development of immunotherapy holds enormous promise to escalate the efficacy of cancer therapy. Understanding better immune mechanisms and tumor biology presents, in the future of oncology, an especially wonderful positioning for benefiting from these developments to result in more effective, selective, and tailored therapeutic options for patients suffering from cancer. With continued research in these fields, we can anticipate seeing significant advancements in cancer diagnosis, treatment, and prevention.

## References

[1] Cleanclay W. D., Zakari S., Adigun T. O. (2023). Cancer Biology and Therapeutics: Navigating Recent Advances and Charting Future Directions. *Tropical Journal of Natural Product Research*.

[2] Cancer Research UK (2015). Worldwide Cancer Statistics. https://www.cancerresearchuk.org/health-professional/cancer-statistics/worldwide-cancer.

[3] Ciardiello F., Tortora G. (2001). A Novel Approach in the Treatment of Cancer: Targeting the Epidermal Growth Factor Receptor. *Clinical Cancer Research: An Official Journal of the American Association for Cancer Research*.

[4] Targeted Therapy for Cancer—NCI (nciglobal, ncienterprise) (2014). https://www.cancer.gov/about-cancer/treatment/types/targeted-therapies.

[5] Pucci C., Martinelli C., Ciofani G. (2019). Innovative Approaches for Cancer Treatment: Current Perspectives and New Challenges. *Ecancermedicalscience*.

[6] Ventola C. L. (2017). Cancer Immunotherapy, Part 1: Current Strategies and Agents. *P T*.

[7] Debela D. T., Muzazu S. G., Heraro K. D. (2021). New Approaches and Procedures for Cancer Treatment: Current Perspectives. *SAGE Open Medicine*.

[8] Enoma D. O., Bishung J., Abiodun T., Ogunlana O., Osamor V. C. (2022). Machine Learning Approaches to Genome-Wide Association Studies. *Journal of King Saud University-Science*.

[9] Brace C. (2011). Thermal Tumor Ablation in Clinical Use. *IEEE Pulse*.

[10] Jhawar S. R., Thandoni A., Bommareddy P. K. (2017). Oncolytic Viruses—Natural and Genetically Engineered Cancer Immunotherapies. *Frontiers in Oncology*.

[11] Kojima Y., Tanaka M., Sasaki M. (2024). Induction of Ferroptosis by Photodynamic Therapy and Enhancement of Antitumor Effect With Ferroptosis Inducers. *Journal of Gastroenterology*.

[12] Lospinoso Severini L., Bufalieri F., Infante P., Di Marcotullio L. (2022). Proteolysis-Targeting Chimera (PROTAC): Is the Technology Looking at the Treatment of Brain Tumors?. *Frontiers in Cell and Developmental Biology*.

[13] Oluwajembola A. M., Cleanclay W. D., Onyia A. F. (2024). Photosensitizers in Photodynamic Therapy: An Advancement in Cancer Treatment. *Results in Chemistry*.

[14] Das S. K., Menezes M. E., Bhatia S. (2015). Gene Therapies for Cancer: Strategies, Challenges and Successes. *Journal of Cellular Physiology*.

[15] Definition of gene therapy—NCI Dictionary of Cancer Terms—NCI (nciglobal, ncienterprise) (2011). https://www.cancer.gov/publications/dictionaries/cancer-terms/def/gene-therapy.

[16] Osamor V., Chinedu S., Azuh D., Iweala E., Ogunlana O. (2016). The Interplay of Post-Translational Modification and Gene Therapy. *Drug Design, Development and Therapy*.

[17] Tian Z., Liang G., Cui K. (2021). Insight Into the Prospects for RNAi Therapy of Cancer. *Frontiers in Pharmacology*.

[18] Ghoncheh M., Pournamdar Z., Salehiniya H. (2016). Incidence and Mortality and Epidemiology of Breast Cancer in the World. *Asian Pacific Journal of Cancer Prevention*.

[19] Dastjerd N. T., Valibeik A., Rahimi Monfared S. (2022). Gene Therapy: A Promising Approach for Breast Cancer Treatment. *Cell Biochemistry and Function*.

[20] Gote V., Pal D. (2021). Octreotide-Targeted Lcn2 siRNA PEGylated Liposomes as a Treatment for Metastatic Breast Cancer. *Bioengineering*.

[21] Sheikh-Hosseini M., Larijani B., Gholipoor Kakroodi Z. (2021). Gene Therapy as an Emerging Therapeutic Approach to Breast Cancer: New Developments and Challenges. *Human Gene Therapy*.

[22] Darvin P., Toor S. M., Sasidharan Nair V., Elkord E. (2018). Immune Checkpoint Inhibitors: Recent Progress and Potential Biomarkers. *Experimental & Molecular Medicine*.

[23] Hu Q., Sun W., Wang J. (2018). Conjugation of Haematopoietic Stem Cells and Platelets Decorated With Anti-PD-1 Antibodies Augments Anti-Leukaemia Efficacy. *Nature Biomedical Engineering*.

[24] Wang Z., Wu Z., Liu Y., Han W. (2017). New Development in CAR-T Cell Therapy. *Journal of Hematology & Oncology*.

[25] Almåsbak H., Aarvak T., Vemuri M. C. (2016). CAR T Cell Therapy: A Game Changer in Cancer Treatment. *Journal of Immunology Research*.

[26] Jogalekar M. P., Rajendran R. L., Khan F., Dmello C., Gangadaran P., Ahn B.-C. (2022). CAR T-Cell-Based Gene Therapy for Cancers: New Perspectives, Challenges, and Clinical Developments. *Frontiers in Immunology*.

[27] Newick K., O’Brien S., Moon E., Albelda S. M. (2017). CAR T Cell Therapy for Solid Tumors. *Annual Review of Medicine*.

[28] Greenbaum U., Mahadeo K. M., Kebriaei P., Shpall E. J., Saini N. Y. (2020). Chimeric Antigen Receptor T-Cells in B-Acute Lymphoblastic Leukemia: State of the Art and Future Directions. *Frontiers in Oncology*.

[29] Narkhede M., Mehta A., Ansell S. M., Goyal G. (2021). CAR T-Cell Therapy in Mature Lymphoid Malignancies: Clinical Opportunities and Challenges. *Annals of Translational Medicine*.

[30] Rendo M. J., Joseph J. J., Phan L. M., DeStefano C. B. (2022). CAR T-Cell Therapy for Patients With Multiple Myeloma: Current Evidence and Challenges. *Blood and Lymphatic Cancer: Targets and Therapy*.

[31] Bhaskar S. T., Dholaria B., Savani B. N., Sengsayadeth S., Oluwole O. (2024). Overview of Approved CAR-T Products and Utility in Clinical Practice. *Clinical Hematology International*.

[32] Wang J.-Y., Wang L. (2023). CAR-T Cell Therapy: Where Are We Now, and Where Are We Heading?. *Blood Science*.

[33] Lindner S. E., Johnson S. M., Brown C. E., Wang L. D. (2020). Chimeric Antigen Receptor Signaling: Functional Consequences and Design Implications. *Science Advances*.

[34] Adkins S. (2019). CAR T-Cell Therapy: Adverse Events and Management. *Journal of the Advanced Practitioner in Oncology*.

[35] Schubert M.-L., Schmitt M., Wang L. (2021). Side-Effect Management of Chimeric Antigen Receptor (CAR) T-Cell Therapy. *Annals of Oncology*.

[36] Ke X., Shen L. (2017). Molecular Targeted Therapy of Cancer: The Progress and Future Prospect. *Frontiers in Laboratory Medicine*.

[37] Padma V. V. (2015). An Overview of Targeted Cancer Therapy. *Biomedicine*.

[38] Gogia P., Ashraf H., Bhasin S., Xu Y. (2023). Antibody–Drug Conjugates: A Review of Approved Drugs and Their Clinical Level of Evidence. *Cancers*.

[39] Mukherjee A., Bandyopadhyay D. (2024). Targeted Therapy in Breast Cancer: Advantages and Advancements of Antibody–Drug Conjugates, a Type of Chemo-Biologic Hybrid Drugs. *Cancers*.

[40] Riccardi F., Dal Bo M., Macor P., Toffoli G. (2023). A Comprehensive Overview on Antibody-Drug Conjugates: From the Conceptualization to Cancer Therapy. *Frontiers in Pharmacology*.

[41] Al-Koussa H., El Mais N., Maalouf H., Abi-Habib R., El-Sibai M. (2020). Arginine Deprivation: A Potential Therapeutic for Cancer Cell Metastasis? A Review. *Cancer Cell International*.

[42] Chu Y.-D., Lai M.-W., Yeh C.-T. (2023). Unlocking the Potential of Arginine Deprivation Therapy: Recent Breakthroughs and Promising Future for Cancer Treatment. *International Journal of Molecular Sciences*.

[43] Kumari N., Bansal S. (2021). Arginine Depriving Enzymes: Applications as Emerging Therapeutics in Cancer Treatment. *Cancer Chemotherapy and Pharmacology*.

[44] Patil M. D., Bhaumik J., Babykutty S., Banerjee U. C., Fukumura D. (2016). Arginine Dependence of Tumor Cells: Targeting a Chink in Cancer’s Armor. *Oncogene*.

[45] Zou S., Wang X., Liu P., Ke C., Xu S. (2019). Arginine Metabolism and Deprivation in Cancer Therapy. *Biomedicine & Pharmacotherapy*.

[46] Feun L., You M., Wu C. (2008). Arginine Deprivation as a Targeted Therapy for Cancer. *Current Pharmaceutical Design*.

[47] Tomlinson B. K., Thomson J. A., Bomalaski J. S. (2015). Phase I Trial of Arginine Deprivation Therapy With ADI-PEG 20 Plus Docetaxel in Patients With Advanced Malignant Solid Tumors. *Clinical Cancer Research*.

[48] Sun N., Zhao X. (2022). Argininosuccinate Synthase 1, Arginine Deprivation Therapy and Cancer Management. *Frontiers in Pharmacology*.

[49] Carpentier J., Pavlyk I., Mukherjee U., Hall P. E., Szlosarek P. W. (2022). Arginine Deprivation in SCLC: Mechanisms and Perspectives for Therapy. *Lung Cancer: Targets and Therapy*.

[50] Matos A., Carvalho M., Bicho M., Ribeiro R. (2021). Arginine and Arginases Modulate Metabolism, Tumor Microenvironment and Prostate Cancer Progression. *Nutrients*.

[51] Bowles T. L., Kim R., Galante J. (2008). Pancreatic Cancer Cell Lines Deficient in Argininosuccinate Synthetase Are Sensitive to Arginine Deprivation by Arginine Deiminase. *International Journal of Cancer*.

[52] Liu Y., Shi Y. (2020). Mitochondria as a Target in Cancer Treatment. *MedComm*.

[53] Mukherjee S., Bhatti G. K., Chhabra R., Reddy P. H., Bhatti J. S. (2023). Targeting Mitochondria as a Potential Therapeutic Strategy Against Chemoresistance in Cancer. *Biomedicine & Pharmacotherapy*.

[54] Peng H., Yao F., Zhao J. (2023). Unraveling Mitochondria-Targeting Reactive Oxygen Species Modulation and Their Implementations in Cancer Therapy by Nanomaterials. *Exploration*.

[55] Roth K. G., Mambetsariev I., Kulkarni P., Salgia R. (2020). The Mitochondrion as an Emerging Therapeutic Target in Cancer. *Trends in Molecular Medicine*.

[56] Smith R. A. J., Hartley R. C., Murphy M. P. (2011). Mitochondria-Targeted Small Molecule Therapeutics and Probes. *Antioxidants & Redox Signaling*.

[57] Kalyanaraman B., Cheng G., Hardy M. (2017). Mitochondria-Targeted Metformins: Anti-Tumour and Redox Signalling Mechanisms. *Interface Focus*.

[58] Javadov S., Kozlov A. V., Camara A. K. S. (2020). Mitochondria in Health and Diseases. *Cells*.

[59] Filep J. G. (2022). Targeting Neutrophils for Promoting the Resolution of Inflammation. *Frontiers in Immunology*.

[60] Németh T., Sperandio M., Mócsai A. (2020). Neutrophils as Emerging Therapeutic Targets. *Nature Reviews Drug Discovery*.

[61] Subhan M. A., Torchilin V. P. (2021). Neutrophils as an Emerging Therapeutic Target and Tool for Cancer Therapy. *Life Sciences*.

[62] Xiong S., Dong L., Cheng L. (2021). Neutrophils in Cancer Carcinogenesis and Metastasis. *Journal of Hematology & Oncology*.

[63] Wu M., Ma M., Tan Z., Zheng H., Liu X. (2020). Neutrophil: A New Player in Metastatic Cancers. *Frontiers in Immunology*.

[64] Bonecchi R., Mantovani A., Jaillon S. (2022). Chemokines as Regulators of Neutrophils: Focus on Tumors, Therapeutic Targeting, and Immunotherapy. *Cancers*.

[65] SenGupta S., Hein L. E., Parent C. A. (2021). The Recruitment of Neutrophils to the Tumor Microenvironment Is Regulated by Multiple Mediators. *Frontiers in Immunology*.

[66] Schepisi G., Gianni C., Cursano M. C. (2023). Immune Checkpoint Inhibitors and Chimeric Antigen Receptor (CAR)-T Cell Therapy: Potential Treatment Options Against Testicular Germ Cell Tumors. *Frontiers in Immunology*.

[67] Gupta S., Gupta S. C., Hunter K. D., Pant A. B. (2020). Immunotherapy: A New Hope for Cancer Patients. *Journal of Oncology*.

[68] Burslem G. M., Crews C. M. (2020). Proteolysis-Targeting Chimeras as Therapeutics and Tools for Biological Discovery. *Cell*.

[69] Khan S., He Y., Zhang X. (2020). PROteolysis TArgeting Chimeras (PROTACs) as Emerging Anticancer Therapeutics. *Oncogene*.

[70] Li X., Pu W., Zheng Q., Ai M., Chen S., Peng Y. (2022). Proteolysis-Targeting Chimeras (PROTACs) in Cancer Therapy. *Molecular Cancer*.

[71] Yao T., Xiao H., Wang H., Xu X. (2022). Recent Advances in PROTACs for Drug Targeted Protein Research. *International Journal of Molecular Sciences*.

[72] Zhou X., Dong R., Zhang J.-Y., Zheng X., Sun L.-P. (2020). PROTAC: A Promising Technology for Cancer Treatment. *European Journal of Medicinal Chemistry*.

[73] Sakamoto K. M. (2010). Protacs for Treatment of Cancer. *Pediatric Research*.

[74] Burke M. R., Smith A. R., Zheng G. (2022). Overcoming Cancer Drug Resistance Utilizing PROTAC Technology. *Frontiers in Cell and Developmental Biology*.

[75] Wang Z., Tan M., Su W. (2023). Persistent Degradation of HER2 Protein by Hybrid NanoPROTAC for Programmed Cell Death. *Journal of Medicinal Chemistry*.

[76] Haleem A., Javaid M., Singh R. P., Rab S., Suman R. (2023). Applications of Nanotechnology in Medical Field: A Brief Review. *Global Health Journal*.

[77] Bhatia S. N., Chen X., Dobrovolskaia M. A., Lammers T. (2022). Cancer Nanomedicine. *Nature Reviews Cancer*.

[78] Alhaj-Suliman S. O., Wafa E. I., Salem A. K. (2022). Engineering Nanosystems to Overcome Barriers to Cancer Diagnosis and Treatment. *Advanced Drug Delivery Reviews*.

[79] Soni A., Bhandari M. P., Tripathi G. K. (2023). Nano-Biotechnology in Tumour and Cancerous Disease: A Perspective Review. *Journal of Cellular and Molecular Medicine*.

[80] Xie X. (2023). Application of Nanomedicine in Diagnostic Technology. *Highlights in Science, Engineering and Technology*.

[81] Zhou J., Chen L., Chen L., Zeng X., Zhang Y., Yuan Y. (2022). Emerging Role of Nanoparticles in the Diagnostic Imaging of Gastrointestinal Cancer. *Seminars in Cancer Biology*.

[82] Atapour A., Khajehzadeh H., Shafie M. (2022). Gold Nanoparticle-Based Aptasensors: A Promising Perspective for Early-Stage Detection of Cancer Biomarkers. *Materials Today Communications*.

[83] Li C.-H., Chan M.-H., Chang Y.-C., Hsiao M. (2023). Gold Nanoparticles as a Biosensor for Cancer Biomarker Determination. *Molecules*.

[84] Aiello A., Giannessi F., Percario Z. A., Affabris E. (2020). An Emerging Interplay Between Extracellular Vesicles and Cytokines. *Cytokine & Growth Factor Reviews*.

[85] Priedols M., Paidere G., Santos C. B. (2023). Bifurcated Asymmetric Field Flow Fractionation of Nanoparticles in PDMS-Free Microfluidic Devices for Applications in Label-Free Extracellular Vesicle Separation. *Polymers*.

[86] Hanjani N. A., Esmaelizad N., Zanganeh S. (2022). Emerging Role of Exosomes as Biomarkers in Cancer Treatment and Diagnosis. *Critical Reviews in Oncology/Hematology*.

[87] Pirisinu M., Pham T. C., Zhang D. X., Hong T. N., Nguyen L. T., Le M. T. (2022). Extracellular Vesicles as Natural Therapeutic Agents and Innate Drug Delivery Systems for Cancer Treatment: Recent Advances, Current Obstacles, and Challenges for Clinical Translation. *Seminars in Cancer Biology*.

[88] Saleem T., Sumrin A., Bilal M., Bashir H., Khawar M. B. (2022). Tumor-Derived Extracellular Vesicles: Potential Tool for Cancer diagnosis, Prognosis, and Therapy. *Saudi Journal of Biological Sciences*.

[89] Choi H., Yim H., Park C. (2022). Targeted Delivery of Exosomes Armed With Anti-Cancer Therapeutics. *Membranes*.

[90] Moon B., Chang S. (2022). Exosome as a Delivery Vehicle for Cancer Therapy. *Cells*.

[91] Wu M., Wang M., Jia H., Wu P. (2022). Extracellular Vesicles: Emerging Anti-Cancer Drugs and Advanced Functionalization Platforms for Cancer Therapy. *Drug Delivery*.

[92] Vergani E., Daveri E., Vallacchi V. (2022). Extracellular Vesicles in Anti-Tumor Immunity. *Seminars in Cancer Biology*.

[93] Bera K., Braman N., Gupta A., Velcheti V., Madabhushi A. (2022). Predicting Cancer Outcomes With Radiomics and Artificial Intelligence in Radiology. *Nature Reviews Clinical Oncology*.

[94] Ye D.-M., Wang H.-T., Yu T. (2020). The Application of Radiomics in Breast MRI: A Review. *Technology in Cancer Research & Treatment*.

[95] Lu Y., Chan Y.-T., Tan H.-Y., Li S., Wang N., Feng Y. (2020). Epigenetic Regulation in Human Cancer: The Potential Role of Epi-Drug in Cancer Therapy. *Molecular Cancer*.

[96] Ma R., Li Z., Chiocca E. A., Caligiuri M. A., Yu J. (2023). The Emerging Field of Oncolytic Virus-Based Cancer Immunotherapy. *Trends in Cancer*.

[97] Andtbacka R. H. I., Kaufman H. L., Collichio F. (2015). Talimogene Laherparepvec Improves Durable Response Rate in Patients With Advanced Melanoma. *Journal of Clinical Oncology*.

[98] Ribas A., Dummer R., Puzanov I. (2017). Oncolytic Virotherapy Promotes Intratumoral T Cell Infiltration and Improves Anti-PD-1 Immunotherapy. *Cell*.

[99] Lang F. F., Conrad C., Gomez-Manzano C. (2018). Phase I Study of DNX-2401 (Delta-24-RGD) Oncolytic Adenovirus: Replication and Immunotherapeutic Effects in Recurrent Malignant Glioma. *Journal of Clinical Oncology*.

[100] Hardcastle J., Kurozumi K., Dmitrieva N. (2010). Enhanced Antitumor Efficacy of Vasculostatin (Vstat120) Expressing Oncolytic HSV-1. *Molecular Therapy*.

[101] Breitbach C. J., Burke J., Jonker D. (2011). Intravenous Delivery of a Multi-Mechanistic Cancer-Targeted Oncolytic Poxvirus in Humans. *Nature*.

[102] Hunter B., Hindocha S., Lee R. W. (2022). The Role of Artificial Intelligence in Early Cancer Diagnosis. *Cancers*.

[103] Koh D.-M., Papanikolaou N., Bick U. (2022). Artificial Intelligence and Machine Learning in Cancer Imaging. *Communications Medicine*.

[104] Sufyan M., Shokat Z., Ashfaq U. A. (2023). Artificial Intelligence in Cancer Diagnosis and Therapy: Current Status and Future Perspective. *Computers in Biology and Medicine*.

[105] Goldenberg S. L., Nir G., Salcudean S. E. (2019). A New Era: Artificial Intelligence and Machine Learning in Prostate Cancer. *Nature Reviews Urology*.

[106] Yoo S.-K., Fitzgerald C. W., Cho B. A. (2025). Prediction of Checkpoint Inhibitor Immunotherapy Efficacy for Cancer Using Routine Blood Tests and Clinical Data. *Nature Medicine*.

[107] Tătaru O. S., Vartolomei M. D., Rassweiler J. J. (2021). Artificial Intelligence and Machine Learning in Prostate Cancer Patient Management—Current Trends and Future Perspectives. *Diagnostics*.

[108] Bhattacharya I., Khandwala Y. S., Vesal S. (2022). A Review of Artificial Intelligence in Prostate Cancer Detection on Imaging. *Therapeutic Advances in Urology*.

[109] Ali S., Rehman M. U., Yatoo A. M., Arafah A., Khan A., Rashid S. (2023). TGF-β Signaling Pathway: Therapeutic Targeting and Potential for Anti-Cancer Immunity. *European Journal of Pharmacology*.

[110] He C. C., Lin D. M., Liu H. Z., Wang F. F., Guo X. F., Zhang X. B. (2023). Nonpharmacological Interventions for Management of the Pain-Fatigue-Sleep Disturbance Symptom Cluster in Breast Cancer Patients: A Systematic Review and Network Meta-Analysis of Randomized Controlled Trials. *Journal of Pain Research*.

[111] Srivastava R. (2025). Advancing Precision Oncology with AI-Powered Genomic Analysis. *Frontiers in Pharmacology*.

[112] Alnasser S. M. (2022). Stem Cell Challenge in Cancer Progression, Oncology and Therapy. *Gene*.

[113] Chu D.-T., Nguyen T. T., Tien N. L. B. (2020). Recent Progress of Stem Cell Therapy in Cancer Treatment: Molecular Mechanisms and Potential Applications. *Cells*.

[114] Yang L., Shi P., Zhao G. (2020). Targeting Cancer Stem Cell Pathways for Cancer Therapy. *Signal Transduction and Targeted Therapy*.

[115] Bao B., Ahmad A., Azmi A. S., Ali S., Sarkar F. H. (2013). Overview of Cancer Stem Cells (CSCs) and Mechanisms of Their Regulation: Implications for Cancer Therapy. *Current Protocols in Pharmacology*.

[116] Jubelin C., Muñoz-Garcia J., Cochonneau D., Moranton E., Heymann M.-F., Heymann D. (2022). Biological Evidence of Cancer Stem-Like Cells and Recurrent Disease in Osteosarcoma. *Cancer Drug Resistance*.

[117] Liang L., Kaufmann A. M. (2023). The Significance of Cancer Stem Cells and Epithelial–Mesenchymal Transition in Metastasis and Anti-Cancer Therapy. *International Journal of Molecular Sciences*.

[118] Bagó J. R., Sheets K. T., Hingtgen S. D. (2016). Neural Stem Cell Therapy for Cancer. *Methods*.

[119] Karnoub A. E., Dash A. B., Vo A. P., Sullivan A., Brooks M. W., Bell G. W. (2007). Mesenchymal Stem Cells Within Tumour Stroma Promote Breast Cancer Metastasis. *Nature*.

[120] Martin L., Watanabe S., Fainsinger R., Lau F., Ghosh S., Quan H. (2010). Prognostic Factors in Patients With Advanced Cancer: Use of the Patient-Generated Subjective Global Assessment in Survival Prediction. *Journal of Clinical Oncology*.

[121] Ramasamy R., Lam E. W. F., Soeiro I., Tisato V., Bonnet D., Dazzi F. (2007). Mesenchymal Stem Cells Inhibit Proliferation and Apoptosis of Tumor Cells: Impact on in Vivo Tumor Growth. *Leukemia*.

